# The AFFIRM Model for Navigating Challenges in Continuously‐Interfaith and Divergent‐Interfaith Marriages

**DOI:** 10.1111/famp.70196

**Published:** 2026-09-17

**Authors:** Justin J. Hendricks, David C. Dollahite, Loren D. Marks, Sam A. Hardy, Chelsea Johnson

**Affiliations:** ^1^ The Pennsylvania State University University Park Pennsylvania USA; ^2^ Brigham Young University Provo Utah USA; ^3^ Private Marriage and Family Therapy Practice St George Utah USA

**Keywords:** divergent‐interfaith, interfaith, qualitative, religion and family, religious heterogeneity

## Abstract

The primary purpose of this study was to identify the processes that helped happy and stable interfaith marriages navigate religious differences. To contextualize these adaptive processes, we secondarily explored the interfaith challenges these couples experienced. A final exploratory aim compared these challenges and processes in continuously‐interfaith couples who started as and remained interfaith, and divergent‐interfaith couples, an understudied subgroup who initially shared a religion but later became interfaith due to religious changes. In‐depth interviews were conducted with 30 interfaith couples (*N* = 60) that had been recruited through purposive sampling who reported being happily married on average 18.7 years. Six core themes were identified through team‐based systematic thematic analysis using NVivo. Many couples reported several significant challenges because of their religious differences, as discussed in our first theme. We organized the five themes regarding how couples navigated religious differences to create the AFFIRM model. That is, the adaptive processes we identified in these interfaith couples were that they (a) Assisted and supported each other's practices and beliefs, (b) Focused on similarities of belief, (c) Fostered religious Intimacy and understanding, (d) Respected each other's religious choices, and (e) made Marriage their first and highest priority. Themes are discussed in terms of continuously‐interfaith couples (*n* = 25) and divergent‐interfaith couples (*n* = 5). Interfaith couples may benefit from the progressive addition of the AFFIRM strategies in treatment. Replication of these qualitative findings with other interfaith couples are indicated to refine usefulness of the model.

## 1 Introduction

Interfaith marriage has risen markedly, with United States (US) couples married since 2010 twice as likely to marry across faiths as those married in the 1950s and 1960s (Cox 2022; Pew Research Center 2015). Interfaith marriage, and its other names including mixed‐faith marriage, interreligious marriage, or denominational heterogamy, refers to differences in religious identification between spouses, though these couples often also have religious differences in belief or practice (Stanford et al. 2025). Among couples married since 2010, 32%–39% are interfaith, split almost equally between (a) religious‐secular marriages and (b) interfaith marriages with spouses from two different religious traditions (Cox 2022; Pew Research Center 2015). Notably, religious‐secular marriages have grown from 5% before 1960 to 15%–18% since 2010, driven by the rise of religiously unaffiliated individuals.

Although there may be some benefits to interfaith marriages, such as increased acceptance and tolerance (Colaner et al. 2023; Shoaf et al. 2022), studies consistently find that interfaith couples report higher divorce rates and lower marital quality (Hwang et al. 2019; Riley 2013). Challenges faced by interfaith couples include contextual factors, such as a lack of community and family support, as well as marital and parenting factors like difficulties in accommodating different religious beliefs and practices (Reiter and Gee 2008) and disagreements over how to raise children religiously (Riley 2013; Walsh 2010). Even though interfaith couples constitute a growing demographic and many face challenges in their relationships, limited research has been conducted to explore the strategies that such couples report to help them navigate religious differences and build a fulfilling, enduring marriage. Further, in this time of religious and political polarization, understanding how couples with significant religious differences can make their marriage work may hold transferable implications for society at large.

Therefore, the current study qualitatively explored how interfaith couples who self‐reported being in a happy and stable interfaith marriage have successfully navigated their religious differences. To contextualize these adaptive processes, we secondarily explored the challenges arising from religious differences that these couples experienced. Building on this, a secondary exploratory aim compares these processes in continuously‐interfaith couples who started as and remained interfaith, and divergent‐interfaith couples, an understudied subgroup who initially shared a religion but later became interfaith due to religious changes. Cheney and Priest (2025) applied Family Systems Theory to show how religious divergence can destabilize the marital “triangle” once anchored in shared beliefs, requiring couples to reconstruct new foundations or risk relational disintegration. They suggest that divergent‐interfaith couples may face distinct challenges and unique adaptive tasks from continuously‐interfaith couples, as they must renegotiate not only religious differences but also the loss of previously shared foundations. Accordingly, the present study examines marital challenges and the processes that promote stability and flourishing in both continuously‐interfaith and divergent‐interfaith marriages.

### 1.1 Potential Challenges of Being in an Interfaith Marriage

Interfaith marriages present unique relational dynamics and challenges (Hwang et al. 2021). However, these challenges do not solely define interfaith relationships, nor do they diminish the strengths these couples can develop through their experiences (Colaner et al. 2023; Shoaf et al. 2022). Understanding these challenges and their underlying causes is crucial for supporting interfaith couples and identifying gaps in existing research.

Consistent with the framework of homogamy, religious differences are consistently linked to increased marital challenges throughout the life course (Hwang et al. 2021; Schafer and Kwon 2019; Schramm et al. 2012). Interfaith couples considering marriage have reported difficulties in deciding how faith will feature in their wedding and feeling ostracized from faith communities (Riley 2013). Denominational differences are associated with lower marital adjustment for newly married couples in their first marriages, but interestingly not for remarriages (Schramm et al. 2012). Hwang et al. (2021) found that interfaith couples started with lower marital satisfaction compared to shared‐faith couples and stayed lower over the 17‐year duration of the study. Additionally, older couples with differing religious affiliations report lower relationship happiness, emphasizing the enduring challenges interfaith couples face (Schafer and Kwon 2019).

Interfaith marriages are more likely to end in divorce than shared‐faith marriages (Hwang et al. 2019; Riley 2013). However, some interfaith combinations, such as Catholic‐Mainline Protestant marriages, have a lower divorce risk (Riley 2013). Interfaith couples face higher divorce risks when at least one partner, especially the wife, belongs to an exclusivist religion that often emphasizes exclusive possession of religious truth, strong boundary maintenance, and marriage within the faith (e.g., fundamentalist, evangelical, Latter‐day Saint; Lehrer and Chiswick 1993; Vaaler et al. 2009). Relatedly, marriages with at least one religious partner, even in interfaith combinations, report higher marital satisfaction and lower marital dissolution than shared secular marriages where neither partner is religious (Boulis and Torgler 2024; Lee and Lee 2023). Lehrer's (2009) study comparing divorce rates at the 5‐year mark indicated that Latter‐day Saint or Mormon^1^ couples experienced the largest differences between shared‐faith and interfaith couples. While only 13% of shared‐faith Latter‐day Saint couples divorced, the interfaith (LDS with other) divorce rate was 40%. Exploring happy and stable interfaith combinations at higher risk for divorce (e.g., LDS with other) may prove particularly insightful in understanding adaptive interfaith processes.

Interfaith couples report higher religious conflict, attributing it to differences in beliefs and practices (Reiter and Gee 2008), and affiliation differences are more likely to be linked to lower marital outcomes combined with other religious differences (Riley 2013). Religious differences are also associated with increased conflict over finances and household labor (Curtis and Ellison 2002). US interfaith couples are more likely to experience religious disagreements than shared‐faith couples, with 32% of religious‐secular marriages and 28% of interfaith couples reporting such conflicts at least some of the time, compared to 19% of shared‐faith couples (Pew Research Center 2016).

One of the most significant challenges interfaith parents face is deciding how to raise their children religiously. Riley (2013) highlights this as “probably the highest hurdle interfaith parents face” (p. 80), with only about 38% of interfaith parents having adult children who identify with their specific faith (Bengtson et al. 2013; Pew Research Center 2016). In sum, previous findings help paint the picture of the problems interfaith couples can face, why they may face them, and indicate the need for assistance.

While challenges have been primarily examined in interfaith couples as a whole, one subgroup that has received little empirical attention is divergent‐interfaith couples. Qualitative research indicates that divergent‐interfaith couples struggle to balance religious and relational loyalties (Coffee et al. 2021). Zaloudek (2005) found that after diverging, her sample (*n* = 4 couples) experienced conflict, loss of common interests, and considered separation, but eventually reported positive effects from the religious/spiritual change. Some prior research has explored how continuously‐interfaith and divergent‐interfaith couples navigate religious differences, as discussed next.

### 1.2 Previous Findings on How to Navigate Religious Differences

Despite the challenges interfaith marriages sometimes present, relatively few studies have systematically explored how they navigate religious differences. However, some research has identified characteristics and processes that promote higher marital quality and stability. Colaner et al. (2023) conducted a systematic review of research literature from 2000 to 2022 on effective communication strategies employed by interfaith and multiethnic‐racial families to handle differences among family members. The review outlined four key processes documented in the literature regarding interfaith families: (a) constructive conflict, (b) open communication, (c) accommodative communication, and (d) religious pluralism. However, it is important to note that Colaner et al.'s (2023) review did not focus exclusively on addressing marital religious differences and was heavily informed by research on parent–child religious differences.

According to Parsons et al. (2007), individuals in interfaith marriages who maintained a strong sense of identity and successfully balanced individuality with togetherness (i.e., differentiation) experienced greater marital satisfaction. Supporting a spouse's religious/spiritual beliefs and practices has been linked to lower relational distress (Reiter and Gee 2008). Hughes and Dickson (2005) found that constructive communication during religious disagreements, characterized by mutual discussion, emotional expression, and meaningful problem‐solving rather than avoidance or withdrawal, was the strongest predictor of marital satisfaction. Hughes (2004) also observed that interfaith partners with an intrinsic religious orientation were more likely to engage in constructive communication. Lastly, women's—but not men's—greater willingness to tolerate religious differences with their spouses provided greater marital protection (Hwang et al. 2019).

Even fewer studies have examined adaptive marital processes in divergent‐interfaith couples, with five notable exceptions all using small qualitative samples. Coffee et al. (2021) and Story Chavez et al. (2026) reported these couples benefited when they prioritized their relationship as equally important or more important than religion. Zaloudek (2005) identified accommodation and acceptance of difference as factors that helped relationships improve after initial challenges from diverging faiths. Similarly, Knight et al. (2019) observed that deep acceptance of changes and forming a new family identity helped families and couples adapt successfully. Story Chavez et al. (2026) also pointed to the importance of differentiation and respect. Zimmerman et al. (2015) highlighted the importance of flexibility in facilitating adaptive relational outcomes. Although these studies suggest potential adaptive processes, each has limited generalizability, and two included nonmarital relationships (e.g., parent–child; Knight et al. 2019; Zimmerman et al. 2015).

Given the limited research in this area, more work is needed to explore and understand positive marital processes in divergent‐interfaith marriages and interfaith couples more broadly. This nascent field requires further exploratory studies, for which qualitative methods are particularly well‐suited to identify adaptive relational processes. Additionally, the model of religious dualities highlights religion's capacity to both create and resolve relational struggles (Dollahite et al. 2018). Although research often portrays religion as divisive in interfaith relationships, this model of dualities suggests it may also help address relational challenges in interfaith marriages, a possibility that warrants further study. Given the noted state of the literature, we qualitatively explored the adaptive processes and challenges of religious differences in both continuously‐interfaith and divergent‐interfaith couples.

## 2 Method

### 2.1 Recruitment and Sample Demographics

We used purposive sampling to recruit 30 interfaith couples (*N* = 60), focusing on happy and stable marriages to enable in‐depth exploration of adaptive relational processes (Daly 2007; Dollahite et al. 2023; Marks 2015). Recruitment criteria included couples who self‐reported (a) being part of a happy and stable interfaith marriage, (b) raised or were raising at least one child together, and (c) at least one partner was highly religious. Over 80% of couples were recruited by contacting religious or interfaith groups, leaders, and organizations across the United States primarily via Facebook or email (see Supporting Information for a list of 143 contacts). Communications included study details and participation criteria, requesting referrals who could then contact us if interested. Additional participants, particularly from minority groups (e.g., Black, Hispanic, Jewish), were recruited through additional purposive and convenience sampling, including referrals from research assistants, a prior related study, and Qualtrics panels. Prior to the study, approval was granted by the Brigham Young University Institutional Review Board (ID# F17231).

Participants had been married on average approximately 18.7 years, ranged in age from 31 to 75 (Husband *M* = 49.5, SD = 13.1, Wife *M* = 47.5, SD = 14.1), and averaged three children per couple. Couples were interviewed from 14 states and Washington D.C., representing seven of the eight socio‐religious regions of the United States (Silk and Walsh 2008), with New England being the only unrepresented region. The sample was 63.3% White and 25% racial and ethnic minorities (8.3% Asian, 6.7% Black or African American, 6.7% Hispanic or Latino, and 3.3% Middle Eastern or North African), with race/ethnicity data missing for seven participants (11.7%). Additionally, seven couples were in cross‐cultural or cross‐racial relationships. Educational attainment ranged from high school to Ph.D., with the median being a 4‐year college degree.

Potential recruitment contacts were selected to facilitate geographic and religious diversity, and to oversample key religious minorities (e.g., Judaism, Islam). Qualitative research prioritizes purposive or transferrable sampling over representativeness, thus although major religious groups are underrepresented relative to their portion of the population, the sample included meaningful proportions of Protestants (15.0%, including Black Protestant,^2^ Mainline Protestant, and Evangelical denominations), Catholics (18.3%), and the religiously unaffiliated (18.3%, including Atheists, Agnostics, and Nothing in particular). US minority traditions were overrepresented, particularly Latter‐day Saints (25.0%), Judaism (11.7%), and Islam (6.7%), with smaller groups such as Buddhism (1.7%), Russian Orthodox (1.7%), and mixed religious identities (1.7%) also represented. The notable oversampling of LDS participants was unintended, with LDS‐affiliated groups accounting for only seven of the 143 recruitment contacts (compared to 20 Jewish‐related contacts). The oversample was likely influenced by some of the researchers' affiliation with Brigham Young University, a religious university sponsored by The Church of Jesus Christ of Latter‐day Saints, which may have increased participation willingness among LDS individuals. Although unintended, this oversample offers unique insights into our research questions, as LDS families place particularly high value on marrying within the faith and exhibit some of the strongest disparities in relationship stability between same‐faith and interfaith unions (Lehrer and Chiswick 1993; Riley 2013).

Five of the couples were divergent‐interfaith, meaning that, when initially married, both partners were committed to a shared faith until one partner left or changed faiths (four left religion altogether, and one switched religions). Couples reported that divergence in faith occurred, on average, 8 years prior to the interview (range = 3.5–12 years), with all couples stating that religious differences were harder immediately following the change, but that the relationship adapted over time to become happy and stable. The remaining 25 couples were continuously‐interfaith throughout their marriage. Finally, participants reported that time spent on religious activities ranged from 0 to 8 h/week (median = 2 h) and that donations to religious organizations ranged from zero to 10% of their income (median = 2%).

### 2.2 Data Collection

Before sampling, university institutional review board approval was obtained, and all couples digitally gave their informed consent before being interviewed. Couples were asked 25 semi‐structured interview questions (see Table S1) on dating, marriage, parenting, and faith. Relevant questions to the present article included: “How many of the conflicts in your marriage are due directly or indirectly to religious differences between you and what have you done to manage those conflicts specifically?” “Were there any unforeseen obstacles you had to overcome? And if so, how did you overcome those challenges?” “What would you tell someone else who is entering into an interfaith marriage?” These web‐based (e.g., Skype, Facetime) interviews were conducted by trained graduate and advanced undergraduate students, transcribed verbatim, and rechecked against audio recordings. Participants received a $35 online gift certificate. Interviews were primarily conducted during 2018–2019 and lasted 35.4–186.5 min (*M* = 92.4 min; SD = 34.6 min). After gathering data from a diverse range of couples, the researchers analyzed the interview text.

### 2.3 Analysis

We utilized a four‐phased analysis process with thematic and systematic team‐based coding strategies in NVivo (Allsop et al. 2022; Bernard et al. 2016; Marks 2015). Allsop et al. (2022) recommend using open coding to identify broad themes and then, if applicable, conducting a focused analysis of selected themes and identified quotes to address specific research questions. To help ensure rigor in this process, Bernard et al. (2016) recommend and outline best practices for developing a codebook with inclusion and exclusion criteria to categorize quotes, and Marks (2015) identify best practices for analyzing data as a team. Our four‐phased approach thus alternated between (a) open coding and exploring themes, and (b) codebook‐coding, systematically identifying quotes relevant to each theme and resolving coding differences. The first two phases used each entire interview as the source of analysis, whereas the last two phases analyzed only the responses identified as relevant to the current study. Diversity in coders was prioritized, including both male and female coders, with various religious backgrounds and experiences in shared‐faith and interfaith families.

In Phase 1, the first author and a research assistant performed open coding until reaching theoretical saturation and then created a codebook to classify the identified themes (Allsop et al. 2022). One of these themes, “Navigating an Interfaith Marriage,” became the focal point of the current study and encompassed the experience of navigating marital religious differences and its associated challenges and processes. In Phase 2, eight undergraduate and graduate research assistants, working in pairs, used the codebook to identify text passages for this and other themes. A total of 420 narratives, drawn from all 30 interviews, were coded for this theme and served as the data source for subsequent analysis. Each narrative included shared stories or discussions on the topic, often featuring multiple exchanges between the husband and wife.

In Phase 3, two research assistants independently open‐coded these 420 narratives, identifying themes related to navigating marital religious differences. Based on the frequency and saliency of codes, they created a refined codebook for the top six themes. In Phase 4, to ensure the validity of themes, the coders independently applied the refined codebook, meeting regularly to discuss references, achieving an inter‐rater reliability above 0.90 (Allsop et al. 2022). Based on the resulting numeric content analysis (NCA) and saliency, the coders agreed upon the six main themes (see Table 1) presented in this article, one regarding interfaith challenges and five regarding adaptive marital processes. Exploratory statistical comparisons between continuously‐ and divergent‐interfaith couples are reported in the Supporting Information; due to data limitations from the sample size and space restrictions, these analyses are not discussed in the main text. Frequency of themes for continuous‐interfaith and divergent‐interfaith couples separately is listed in Table S2.

**TABLE 1:** Numeric content analysis of themes.

Overall coding frequencies		
Theme	No. of coding references	No. of couples (%)
Marital challenges in navigating religious differences	107	20 (67%)
A. Assist and support each other's practices and beliefs	143	28 (93%)
F. Focus on similarities of belief	129	29 (97%)
F.I. Foster religious intimacy and understanding	94	24 (80%)
R. Respect each other's religious choices	126	26 (87%)
M. Make marriage the first and highest priority	21	9 (30%)
# Total	420^a^	30

^a^ The sum of the coded references is 620 (not 420) due to some references applying to multiple themes.

## 3 Results

In our analyses, we identified five key processes that interfaith couples reported as being important to their marital quality and stability. To present the findings clearly, we organized the themes into the acronym AFFIRM: (a) **A**ssisted and supported each other's practices and beliefs, (b) **F**ocused on similarities of belief, (c) **F**ostered religious **I**ntimacy and understanding, (d) **R**espected each other's religious choices, and (e) made **M**arriage their first and highest priority. Nine couples reported all five aspects of the AFFIRM model, 14 reported four, and seven reported three or fewer. We do not suggest these couples intentionally followed the AFFIRM model, but rather use it as a conceptual framework to describe the processes couples engaged in. The report includes over 30 verbatim participant quotes to highlight their voices and ground the findings in their experiences (Levitt et al. 2018; Marks 2015). Before describing the components of the AFFIRM model, we first describe some of the marital challenges participants faced from being in an interfaith marriage.

### 3.1 Marital Challenges in Navigating Religious Differences

Two thirds of couples interviewed described marital challenges they faced from navigating religious differences, and a total of 107 narratives about challenges were identified across all interviews. James,^3^ a Catholic husband, said, “The greatest challenge to my marriage is the difference of our religions. … [For]^4^ any Catholic and Mormon having their sights on getting married, I would not recommend it.” For some, challenges stemmed from tensions over differing beliefs and practices or feeling held back from fully engaging in one's faith, as illustrated by the following exchange between Elder and Thelma, who are Baptist and Catholic respectively:

Elder: I been trying to get her for *years* to understand, she can go to her church, and I can go to my church. … Her thing is you've got to go to church together to be a family; that is not the truth …

Thelma: *Really?*

Elder: No, the Bible says you honor God yourself.

Thelma: Well, we're not going to get into that. It says a family that prays together …

Elder: I believe that part, but I don't have to go to your church to pray together.

Thelma: We *ain't* going to get into that part.

Elder: [*To interviewer*] *So*, you know we've had that discussion before, huh?

Although there were only five divergent‐interfaith couples in the sample, they accounted for just over half of all references to marital challenges (see Table S1). Cole and Rachel, a couple originally LDS before Cole became Agnostic, said:

Cole: My relationship with her parents is not as close as it used to be [before I left the church]. … It put a strain on our [marriage] relationship. … When they first found out I got blacklisted for a little while.

Rachel: (*chuckles*) You still kind of are …

Leah, an LDS wife, and Peter, who was formerly LDS and now is Buddhist, said:

Leah: I remember the night that my husband said, ‘I've never gotten a testimony [that this religion is true]. I was awake that entire night thinking. … It was a rough night. … I felt betrayed for years and years before I really understood that he didn't deliberately betray me. Then I felt remorse and regret that I wasn't safer for him to tell me sooner … I mean there was a period of time in the middle of our marriage that I was adamant [that] my husband is going to get his answer [that this religion is true]. We're going to say our prayer[s] together, going to read scriptures every night. … And it just put such a strain on our relationship and really put a lot of stress on him and a lot of depression and different things. …

Peter: Obviously this faith transition has been an obstacle … we talked about divorce a couple times. … There were definitely some really hard conversations that we had, for me, that made me worry: Is what I'm doing going to lead us there?

In summary, being in an interfaith relationship strained most participants' marriages, though 10 couples did not share any religion‐related conflicts. Despite these difficulties, when invited to share advice for someone entering an interfaith marriage or to describe how they navigated religious conflicts, most couples described ways they were able to mitigate and avoid these challenges, the first of which was to assist and support each other's practices and beliefs.

### 3.2 Assist and Support Each Other's Practices and Beliefs

Almost all couples (28/30 or 93%; 143 references) described how assisting and supporting each other's practices and beliefs helped their marriage. This was manifested in a variety of ways, including couples engaging in both religions' practices, such as attending both church services. Caleb, a Jewish husband married to an Agnostic, said, “It was clear that she was willing to participate in the faith in a very active and direct way … as a couple. That was a really important sign for me that this would be something that would work.” Dawn, a religiously unaffiliated wife (formerly LDS), and her LDS husband Sheldon also illustrated this:

Dawn: I served, and I supported everything they did. I had callings [or responsibilities in the church], … [I] just didn't [have] faith …

Sheldon: It would have been a real disaster if she had decided … “we're not going to go to church, I'm not going to let the kids learn something false,” but she was very supportive.

Additionally, Cassandra, an LDS wife married to a Muslim said,

My husband has been super supportive of my church life, and our son's participation in church life. That's been pretty important to me … And then for religious traditions I mean we celebrate everything, and if it's possible to combine traditions we do.

Assistance and support reportedly helped these marriages, in part by fulfilling spouses' important desire for religion in their lives. Supporting each other often required flexibility, compromise, and recognizing that things will not always go your way. Another related part of what helped couples with supporting each other's beliefs and practices was recognizing the overlap between religions, as discussed next.

### 3.3 Focus on Similarities of Belief

All but one couple emphasized the importance of focusing on similarities of belief (29/30 or 97%; 129 coded references). One Methodist husband named Hans said, “[We] started focusing on the common denominator things, [such as] obviously we agree on Christian principles.” Further, other couples felt that it was beneficial to recognize that differences between them were not so large after all. Spencer and Edith, a Catholic–Jewish couple, said,

Spencer: Maybe, sometimes religious differences don't really exist unless you make them exist.

Edith: I learned about Catholicism, and he's learned about Judaism …

Spencer: In a lot of cases, it's not all differences, there [are] a lot of things in common.

Relatedly, a Black Protestant husband named Aiden said, “I look at all the religions and beliefs [and I focus on] the similarities instead of focusing on the differences, because if you look at the similarities there will be more similarities than differences.”

Others similarly found value in minimizing religious differences, but noted that, if the differences between them were wider, this process might prove a more challenging task. Anthony, a Hispanic Catholic Husband said,

Being as I am Catholic raised, and she is Episcopal raised, they are so close to each other … the differences between the two religions are so minute, as far as we're concerned anyway, not as far as the church is concerned, that it really doesn't affect us much. Now, if she was … something other than Christian, then I think there would be conflict there.

Focusing on similarities did not necessarily mean forgetting about or ignoring differences. Indeed, many couples also reported the importance of understanding key religious differences between each of the partner's faiths and fostering religious and spiritual intimacy.

### 3.4 Foster Religious Intimacy and Understanding

Three quarters of the participants made 94 references to the importance of discussing and understanding religious differences as key to their marriage. Divergent‐interfaith couples, though only 17% of the sample, contributed 34 of the 94 references (36%). Divergent‐interfaith couples emphasized establishing and maintaining religious intimacy and understanding after a partner changed faiths. For both groups, this theme involved openness and learning about each religion. Sebastian, who identified as both LDS and Buddhist and was married to a Jehovah's Witness, said, “It [means] something to know [that religious] part of her because it is important to her that I get to know [it], so I have been open.” Eli, a Jewish husband married to a Catholic, said,

It's not going to work if you change who you are. You know, both parties need to be on the same page, they really need to embrace it. It's not a one‐way street kind of thing, both sides need to understand the other.

This also involved talking through disagreements in beliefs and deciding how to tackle certain religious obstacles. Navesh, a Muslim man married to a Catholic said,

For the most part, I don't think we've had any major obstacles … part of it is also probably because of how much we discussed this stuff. We talked about it a lot … we probably left no stone unturned when we were going into this.

Alphonse, an LDS husband married to a Methodist, said,

Having religious conflicts … leads to a lot of good conversations … I think that's what makes our marriage better, is because we do talk about them. We don't just bottle it up and let it fester, we talk about it and get it out in the open, and then go from there.

In summary, communication and understanding were reportedly essential to the success of participants' interfaith marriages. Openly discussing and learning about each other's religions helped couples navigate and accept differences. Resolving disagreements together strengthened their relationships and built a stronger foundation, and often requiring respect for their partner's beliefs, practices, identity, and choices, as discussed next.

### 3.5 Respect Each Other's Religious Choices

Nearly every couple emphasized respecting each other's religious choices as they navigated religious differences in their marriage (26/30 or 87%; 126 references). This respect was manifest in various ways. Elder, a Baptist husband quoted earlier, showed his respect in recognizing the validity of his wife's religious beliefs and practices:

I believe that each one [of] us serve our God in our own way. … I support the Catholic church and she supports the Baptist church. … I believe that's the thing that binds us together, is that she and I both have a place where we can go and worship the Lord.

This respect also involved forgoing judgment, as illustrated by Omar, a Muslim husband:

Anybody who allows religion to become a divisive element in a relationship, they need to go have their heads examined. … Who in the heck are you to make a judgment call? [What], God empowered me to say that because I'm a Muslim that she is less than I am, or because she's a Christian she's more than I am? … I think it is the height of human conceit that we think that somehow, we, tiny as we are, are in a position to pass God's judgement on somebody else.

In addition to respect and avoiding judgment, couples discussed the important and related process of differentiation. Specifically, by recognizing both partners were acting as individuals, and by focusing on their own conduct and decisions while recognizing, respecting, and accepting differences, couples were better able to successfully navigate their differences.

In response to the question “What advice would you give to someone who was entering into an interfaith marriage?” Esther, a Catholic wife married to a Muslim, said,

Go in with it as something you're gaining an asset rather than a deficit of, “Oh, I have to diminish parts of my identity and cultural identity or religious identity because I have to be less of a Catholic or something, because I'm going into this marriage.”

Similarly, Ingrid, an LDS wife married to Ahmad, her religiously unaffiliated husband that was formerly LDS, said,

We both are pretty strong believers in letting the other person do what they want, and we love and trust each other enough to believe that even if the other person's doing something that we personally don't believe in, that they're doing it because they believe that's the best outcome for them. … So, if you want to leave the church, I believe that you feel like that's the best thing for you to do and … I'll respect that. So, I think this whole idea of respecting the other person's choices and not having to be identical [helps avoid conflict] … [and] I feel like a lot of people really conflate unity with sameness.

For Divergent‐Interfaith couples, this theme was often in the context of respecting and honoring their partner's choice to leave their shared faith. Some couples also described that one thing that allowed them to respect and accept their partner's choices was recognizing that their partner's religious choices did not necessarily keep them from living true to their own beliefs. Thus, differentiation and respect may have helped couples reduce their inner tension between staying loyal and committed to their faith and staying loyal and committed to their partner. Balancing these two commitments was explicitly discussed in our final theme, “make *marriage* the first and highest priority.”

### 3.6 Make Marriage the First and Highest Priority

Although less common than other themes, still nearly one third of couples described the importance of putting marriage as their first priority (9/30 or 30%; 21 coded references). Specifically, participants reported things went better when they did not place their religion above their relationship. Maddie, a Presbyterian wife married to a religiously unaffiliated individual, said,

We were having a fall festival at church, and I left this horrible, messy kitchen for my husband … And when I came back from church, he had had it all cleaned up. And I just thought, you know, God gave me a family, a husband first and then children, and if I can't manage what's inside my home, I should not be overextending myself at church.

In balancing religious and relational commitments, some described how their beliefs helped prioritize their relationships. Although divergent‐interfaith couples comprised only 17% of the sample, they accounted for 67% of references to prioritizing marriage. Thus, navigating these tensions may be particularly important for divergent‐interfaith couples. Sheldon, an LDS husband quoted earlier (married to Dawn who had become religiously unaffiliated) said:

My personal beliefs are that the family is the most important thing. … If, for example, it came between me having to decide to stay married, or go to church, I'd choose to stay married. … When we first had our religious separation [and my wife left our church] … I assumed I had to get divorced. … [But] when I started looking for justification and all the support that I felt I would need to justify leaving my wife, it started feeling like the most non‐Christian thing to do.

Despite the challenges that these couples faced, these marriages illustrate that happiness and stability in interfaith marriages are possible, and that these processes helped them achieve that goal. In conclusion, Dennis, a formerly LDS husband that had become religiously unaffiliated, said,

It's been twelve years since I stopped believing. … [She] is very strict and orthodox, and [I have] very passionate feelings to the contrary. … Yes, there are certain difficulties with that and I have certainly experienced that, but I think as long as you can not put yourself first … no matter how strongly you feel about your views and your beliefs, but you are still putting your family first, you can still have a good and healthy family … as long as we're able to not let our beliefs change the level of devotion that we have and commitment to each other and having a healthy marriage.

## 4 Discussion

The purpose of the present study was to understand how couples in happy and stable interfaith marriages navigated religious differences. Our findings indicate that many of these couples faced significant challenges. However, both continuously‐interfaith and divergent‐interfaith couples reported several processes that helped them in navigating their interfaith marriage. We organized and developed a model based on these themes that we called the AFFIRM model, as discussed below. As noted previously, only nine couples reported all five aspects of the AFFIRM model. Thus, it may be possible to achieve a happy and stable interfaith marriage without practicing every one of these processes. However, every couple reported at least one of the five themes in the AFFIRM model, and on average couples reported 3.87 of the five themes.

### 4.1 Assist and Support Each Other's Practices and Beliefs

Our finding regarding the importance of Assisting and supporting each other's practices and beliefs and the need to be flexible in doing so is consistent with prior research noted in the literature review (Reiter and Gee 2008; Zimmerman et al. 2015). Although our study is not generalizable, given the repeated nature of this finding across multiple studies, we tentatively suggest that this process may be transferable to other situations, and that future quantitative studies should be conducted to directly test this recommendation in a larger context.

### 4.2 Focus on Similarities of Belief

The adaptive process of Focusing on similarities of belief and practice supports and extends prior research (Colaner et al. 2023; Hendricks et al. 2024; Shoaf et al. 2022). Although previous literature has highlighted the ability of interfaith couples to build upon religious similarities (Shoaf et al. 2022), the importance of religious pluralism in interfaith marriages (Colaner et al. 2023), and of identifying religious similarities in the parent–child relationship after children leave religion (Hendricks et al. 2024), this study is one of the first to emphasize the role of focusing on religious similarities in strengthening interfaith marriages.

### 4.3 Foster Religious Intimacy and Understanding

The relational benefits from Fostering religious Intimacy and understanding for continuously‐interfaith and divergent‐interfaith couples is a somewhat novel and nuanced finding from our study. Although previous research has highlighted the importance of open communication about religion (Reiter and Gee 2008), constructive conflict in interfaith couples (Colaner et al. 2023), and deep acceptance in divergent‐interfaith families (Knight et al. 2019), we are not aware of prior literature that discusses the importance of religious and spiritual intimacy in interfaith relationships. Spiritual intimacy has been shown elsewhere to be beneficial for marital relationships in general (Kusner et al. 2014), and our finding is consistent with the theory of religious dualities that religion can play a uniting role, not just a dividing role in interfaith relations (Dollahite et al. 2018).

Our study indicates that if both partners are willing, interested, and in a safe space to do so, fostering this type of spiritual intimacy may be possible and beneficial. However, related research on parent–child religious differences suggests that setting boundaries to avoid talking about religious topics can help maintain a healthy parent–child relationship (Worwood et al. 2020). Additionally, McCurry et al. (2012) observed that interfaith couples were less comfortable discussing religion, especially if uncertain about their relationship or ability to talk about spirituality. Future research is needed to explore when limiting religious communication may be more appropriate than seeking religious intimacy.

### 4.4 Respect Each Other's Religious Choices

Similarly, the theme of Respecting each other's religious choices as well as the related process of differentiation noted within this theme both supports and extends previous findings. Previous studies on interfaith families have found that accommodative communication (Colaner et al. 2023) and differentiation (Parsons et al. 2007) are linked to positive relational outcomes. However, Colaner et al. (2023) focused on family religious differences, not just marital religious differences as our study does, and neither study notes the importance of allowing partners to diverge in faith and respecting that choice as these participants described.

### 4.5 Marriage the First and Highest Priority

The recommendation to make marriage the first and highest priority given by our participants echoes the findings of Coffee et al. (2021) and Story Chavez et al. (2026), which found that divergent‐interfaith couples benefited when they prioritized their relationship as equally important or more important than their religion. Given that both the present study and the previous literature discuss this idea primarily in the context of divergent‐interfaith couples, and that both studies are limited small‐sample qualitative projects, future large‐sample quantitative research should be done to examine the importance of this priority. Additionally, couples may benefit from seeing these competing commitments as potentially complementary, as religious commitments often encourage marital commitment and strong families, even in interfaith contexts. Because only 9 of the 30 couples reported intentionally prioritizing marriage in the context of competing religious priorities, this process may be less relevant across the broader interfaith population, though representative research is needed to test this possibility.

### 4.6 Clinical Implications

Spouses entering therapy for interfaith issues vary in emotional intelligence, empathic ability, and motivation to preserve and enhance their marriages. To create a sound treatment plan for interfaith couples, the clinician may assess the clients' goals, their willingness to work toward those goals, and the skills they will need to achieve them. The therapist may also require that clients identify at least one shared goal on which to build joint treatment. We recommend that the AFFIRM strategies provide clinicians with clear treatment goals that are simple for clients to understand. The achievability of these strategies may increase hope and confidence in both clients and clinicians, which research suggests can improve treatment effectiveness (Bartle‐Haring et al. 2022).

In keeping with the prevailing clinical wisdom to pace treatment according to “where the client is” (Goldstein 1983, 267), therapists may wish to consider introducing the AFFIRM strategies according to difficulty level. For example, “fostering religious intimacy and understanding” might be considered the most advanced level since it involves bonding through religion/faith, while “respecting each other's religious choices” and “focusing on common beliefs” allow for bonding around religion/faith. “Assisting and supporting each other's practices and beliefs” might also be regarded as an advanced level since it involves active support, not just passive acceptance.

Attentiveness to Levels 1–3—prioritizing marriage, focusing on commonalities, and respecting differences—may provide enough stability and mutual respect for couples to meaningfully move forward together, even if they do not reach Level 4 of actively supporting differing religious practices or Level 5 of religious intimacy. Like the “Good‐Enough Sex” model (Metz and McCarthy 2007, 351), which seeks to diminish sexual distress in couples by lowering the bar from the idealized to the achievable, treatment of interfaith couples might benefit from the normalization of fulfilling yet less‐than‐ideal outcomes; as with sexual distress, interfaith distress may be partly due to inflated expectations and therefore partly resolvable through recalibrated expectations.

A promising aspect of these strategies is that clients can use them without changing their religious or spiritual convictions. This flexibility reflects John Gottman's long‐standing research showing that even highly satisfied couples often fail to reach consensus on major issues, including religion (Gottman 2000). Gottman's Sound Relationship House model (Gottman et al. 2002) places “Creating Shared Meaning” at the top level, even after “Making Dreams and Aspirations Come True” (p. 389). As discussed previously, bonding through shared meaning, particularly religious/spiritual meaning, is wonderful but difficult to achieve. While this may be an oversimplification of Gottman's model, clients might initially simply focus on making the dreams of each spouse come true and enjoy the deep satisfaction that can accompany such effort.

Similar to Gottman's second step of sharing fondness and admiration, the AFFIRM strategies encourage interfaith spouses to find authentic ways to admire each other, even in early stages of treatment. If they are unable to appreciate each other's religious/spiritual convictions, perhaps they can admire the root values which motivate them to adhere to (or reject) particular practices; for example, one client can value another's devotion to personal integrity and willingness to live according to what they believe is good and true.

Spouses are more likely to appreciate each other when they see one another as having integrity, as opposed to being “brainwashed,” “worldly,” or any of the pejorative labels that are often adopted under the stress of high‐stakes disagreement. Whether discussed explicitly or not, thriving mixed‐faith couples who employ at least some of the AFFIRM strategies may reach consensus on several core values, such as integrity, a personal understanding of truth/goodness, and connection despite differences.

Regardless of the specific values a couple agrees upon, it may be useful for clinicians to encourage such identification. One survey participant suggested that it is a mistake to “conflate unity with sameness,” but the discovery of unifying values/traits can give commonality its due. When spouses believe they lack commonality, they may find each other too foreign to trust, attach to, and bond with, and interfaith couples do not have the luxury of a common belief system that reinforces compatibility.

Even clients who express doubt about their current marriages are often motivated to work on themselves in ways that may improve their flourishing in future romantic partnerships. Clinicians might explain that respecting differences and focusing on commonalities are essential skills for any thriving relationship, so the clients may benefit from practicing them together as long as the marriage lasts. Such an approach may release enough tension and provide enough structure to promote gradual reconciliation in cases where reconciliation seems unachievable to clients. Even when marriages do not endure, treatment may provide enduring benefits to clients and their future relationships.

### 4.7 Limitations and Directions for Future Research

While this study provides valuable insights into navigating religious differences in interfaith marriages, there are some important limitations to note. This study uses a geographically diverse and relatively large sample by qualitative research standards (Marks 2015), but a small sample by quantitative standards (*N* = 60). Moreover, because the sample was purposive and not representative, the findings are not generalizable. The unintended oversampling of Latter‐day Saint participants, while informative, may limit applicability to other interfaith populations. However, as with most qualitative research, the findings are not intended to be generalizable but rather to offer depth and insight that can inform future research and practice. As such, the insights gained from this study may be transferable to similar contexts. Future research may also benefit from exploring how these processes vary by gender, race, and age. Further, though there are some benefits to interviewing couples together, such as spouses filling in each other's gaps and memory lapses and prompting further conversation, interviewing couples together rather than separately could have potentially reduced the transparency of one or both partners. Relatedly, because partner violence or coercion was not assessed, it is unknown how freely the partner with less relational power expressed their views.

Although the subsample of five divergent‐interfaith couples is consistent with prior research, with Story Chavez et al. (2026) interviewing five, Zaloudek (2005) four, and Coffee et al. (2021) seven, the small number of divergent‐interfaith couples limits the ability to draw strong comparisons between couple types. Many prior studies on interfaith marriages likely include divergent‐interfaith couples but, due to methodological limitations such as a lack of longitudinal spousal religious data, have not distinguished this subgroup and instead treated them as traditional interfaith marriages. In this context, although our comparisons remain exploratory, we argue that explicitly identifying and comparing these subgroups rather than collapsing or excluding the smaller divergent‐interfaith subgroup provides a more precise and informative contribution that future research should continue to pursue. Although the greater than threefold overrepresentation in the proportion of reported difficulties might indicate that divergent‐interfaith couples potentially experience greater challenges than continuously‐interfaith marriages, perhaps due to factors such as feeling betrayal, representative quantitative research is needed comparing these groups.

A key strength and weakness is the study's focus on self‐reported happy and stable interfaith marriages. Although this purposive sampling approach provided valuable insights into adaptive marital processes, the findings may not fully capture the experiences of distressed or unsuccessful interfaith marriages, where different strategies may be used or similar strategies may yield different outcomes. Additionally, the reliance on self‐reported marital happiness and retrospective accounts may introduce recall or social desirability bias. Further research on the types of strain in distressed continuous‐ and divergent‐interfaith marriages would be valuable. Lastly, the qualitative nature of the findings limits conclusions about causality or the relative effectiveness of specific AFFIRM processes. Future quantitative research could test the predictive significance of these behaviors or beliefs on marital functioning and outcomes to inform clinical applications.

## 5 Conclusion

The AFFIRM model provides clear and empirically supported suggestions for interfaith couples to help navigate religious differences. Additionally, because many religious couples may experience smaller differences of belief or practice within their faith (Riley 2013), these themes may offer some practical suggestions for how those couples may empathize, heal divisions, or reconcile differences. While the sampling is not generalizable, their alignment with prior research and theory, along with consistency across couples, suggests that many of the processes may be transferrable to other couples in facilitating happy and stable relationships. We hope this study prompts further quantitative research to test the AFFIRM model and other relevant literature in addressing the needs of the growing population of interfaith and divergent‐interfaith couples.

## Funding

This research was supported by funding from the National Institute of Child Health and Human Development (NICHD) to the Population Research Institute at The Pennsylvania State University for Population Research Infrastructure (P2C HD041025) and Social Environments and Population Health Training Grant (T32HD007514). Any opinions, findings, and conclusions or recommendations expressed in this publication are those of the author and do not necessarily reflect the views of the funding agency.

## Conflicts of Interest

The authors declare no conflicts of interest.

## Supporting information


**Table S1:** Interfaith interview questions.
**Table S2:** Frequencies of references made by continuously‐interfaith and divergent‐interfaith couples.
**Table S3:** famp70196‐sup‐0001‐AppendixS1.docx. *T*‐tests of theme frequency between continuously‐interfaith and divergent‐interfaith couples.
**Table S4:** Frequencies of interfaith and divergent‐interfaith couples reporting each theme and results of Fisher's exact test.

## Data Availability

The data that support the findings of this study are available on request from the corresponding author. The data are not publicly available due to privacy or ethical restrictions.

## Endnotes

^1^
“Mormon” is a common but generally non‐preferred term for members of The Church of Jesus Christ of Latter‐day Saints. Therefore, this study uses the terms “Latter‐day Saint” or “LDS.”

^2^
The term “Black Protestant” refers to historically black churches (e.g., African Methodist Episcopal) and is a commonly used classification in distinguishing different traditions within Protestantism (Steensland et al. 2000).

^3^
All participants' names are pseudonyms to protect identity.

^4^
Bracketed words in these quotes are author additions to provide clarity, protect confidentiality, and provide relevant information from other parts of the interview.
