# Supplementary material for: The AFFIRM Model for Navigating Challenges in Continuously‐Interfaith and Divergent‐Interfaith Marriages
Source: Fam Process. 2026 Sep 17;65(3):e70196. doi: 10.1111/famp.70196 (PMC13584837; doi:10.1111/famp.70196)
Supplement: Supplementary file 1 [file FAMP-65-0-s001.docx]

# Supplemental Materials

# The 143 Contacted Religious/Interfaith Groups, Leaders, and Organizations

Advent Christian General Conference, Advent Presbyterian Church, Alabama’s Shule Without Walls, American Baptist Churches USA, American Evangelical Christian Churches, American Muslim, Angelican Province of Christ the King, Antioch Baptist Church, Antiochian Orthodox Christian Archdiocese of North America, Arlington Unitarian Universalists, Asheville Interfaith, Aspiring Mormon Women Discussion, Assemblies of God Church, Assemblies of God USA, Association of Free Lutheran Congregations, Baptist Debate and discussion, Bellevue Baptist Church, Bible Baptist Pastors Sharing Sermons, Bismarck Interfaith, Born Again Friends and Ministers, Briarwood Community Church, Buddha Dharma Education Association Inc., Cathedral of Praise AG, Catholic Faith, Catholic Faith, Apostolic Order, Orthodox Worship, and Evangelical Witness, Center for Faith and Culture, Christian Leadership, Christians of America, Church of the Brethren, Cordova Presbyterian Church, Counsel of Interfaith Families of the United States, Daniel Eisenman (Youtube), East End United Methodist Church Nashville, El-Tawhid Jumah Circle/Unity Mosques, Evangelical and Mormon Interfaith Dialogue, Faith Anglican Church, Cordova, TN, Family of Abraham Nashville, First Assembly Memphis, Friends of East Shore Unitarian Universalists, God First Ministries, Grace Community Church, Greek Orthodox Interfaith Marriage, Heartsong Church, Help and Support for Mixed Couples, Highland Church of Christ, Houston Islam and Interfaith Initiative, Humanistic Judaism, Interfaith Alliance, Interfaith and Community Bulletin Board, Interfaith Center of Greater Philadelphia, Interfaith Community, Interfaith Community for Worship, Interfaith Dialogue, Interfaith Dialogue and Non-Violent Communication, Interfaith Dialogues, Interfaith Families, Interfaith Families of Franklin, MA, Interfaith Families of Greater Philadelphia (Jewish/Christian), Interfaith Families Project of Greater Washington, Interfaith Family, Interfaith Family Philadelphia, Interfaith Fellowship for Peace, Interfaith Forum for Peach on Earth, Interfaith Harmony Council of Bay, Interfaith Liberation Theology, Interfaith NY, Interfaith of Breathitt County, Interfaith Philly, Islamic Center of Nashville, Islamic Judeo-Christian Interfaith Discussion, Jessica Jackley & Reza Aslan (Youtube), Jesus-MyFaith MyWalk Christian Group, Jewish and Christian Alliance for Israel, Jewish Converts and Interfaith Network, Jewish Family Services Interfaith Conversations, Jewish Federation of Nashville and Middle Tennessee, Jewish Multiracial Network, Jewish Muslim Interfaith Families, Jewish/Interfaith Families of Aurora, Jews in All Hues, Kearny Interfaith Network, Largest Christian Group Ever, Living a Christian Life, Meaningful Muslim and Christian Dialogue, MJCCA Interfaith Couples, Momentum Church Memphis, Mormon Blogs, Mormon Discussions, Mormon Mixed-Faith Marriages, Mormons Building Bridges, Muslim Christian InterFaith Families, Muslim Christian Peaceful Dialogue, Muslims for Progressive Values, Muslims for Progressive Values-USA, My Healthy Church- Equipping Spirit Empowered People, Network of Interfaith Family Groups, New Life Interfaith Ministries, North Fulton Interfaith Alliance, One Million Moms Devoted to Christ, One World Family Interfaith Discussion, Owlvine Green (Youtube), Pam Moore (writer on website mazeltogether.org), Pastor to the Pastors, Pastors and Christian Leadership, Planning your Jewish Interfaith Wedding, Preachers in America, Presbyterians Seeking Unity, Protestant, Orthodox, and Catholic Discussion, Rabbi Barry Leff, Temple Beth-El, Alabama, Rabbi John Carrier (Youtube), Reformed Baptist, Religion Debate and Discussion, Religion Discussion, Roman Catholic, Orthodox, and Protestant, Shalom Bayis: for Jewish Interfaith, St. Ann’s Episcopal Church, Nashville, St. Francis of Assisi Catholic Church Memphis, Susan Miller (Author of Being Both: Embracing Two Religions in One Interfaith Family), Temple B’nai Israel, Florence Alabama, Temple Beth Shalom Interfaith Families, Temple Beth-El, Temple Shalom KY, Texans for Interfaith Peace and Justice, The Atheist Voice, The Buddhist, The Chicago Interfaith Family School (Catholic/Jewish), The Christian Muslim Interfaith Bridge, The Church of Jesus Christ of Latter-Day Saints, The Half-Jewish Network, The Interfaith Union, The Love of Christ Church, The Memphis Church, The Temple, The Union School for Interfaith Families (Chicago suburbs, Christian/Jewish), Towson Unitarian Universalist Church, Triangle Interfaith Alliance, Trinity Baptist Church, Unitarian-Universalist Guide for Interfaith Families, United Muslims, UUCT Sharing Unitarian Universalists, Women Preachers, Women’s Interfaith Gatherings, Women’s Interfaith Network.

**Table S1**

*Interfaith Interview Questions*

**Interview Questions**

1. What are some of your beliefs relating to marriage and family?

2. Are there practices or traditions that hold special meaning for you as a couple and/or family?

3. Has your relationship with other important people in your lives influenced your relationship with each other, and if so, how?

4. What do you do to make it through stressful and challenging times?

5. What are the greatest challenges, both external and internal, to your family and marriage being all that you want it to be?

6. All couples have some conflict. Are there ways that your beliefs or practices help avoid or reduce marital conflict?

7. In trying to be a good marital partner and a good parent, from whom or where do you seek guidance?

8. What values or beliefs are most important to the success of your marriage and parenting?

9. As parents, do you strive to share your deepest beliefs with your children? If so, how?

10. Have your deepest beliefs helped you when your kids have struggled for meaning or for guidance?

11. Has your relationship with others influenced your relationship with your children and vice versa has your children’s relationship with others ever influenced their relationship with you?

12. How important is it to you that your children follow in your beliefs?

13. What are your deepest hopes for your children in relation to their future family life?

14. What do you consider to be the most important things for you to do or be as a mother?

15. What do you consider to be the most important things for you to be or do as a father?

16. Did your parents ever comment about inter-faith relationships while you were growing up?

17. How did your parents feel about you marrying outside of your faith?

18. How much did you discuss your differences in faith before you were married and when did you bring up those differences?

19. Did you have conversations before marriage about how religion would be addressed in your home and how you would educate your children. How has that changed?

20. What do your most meaningful conversations with your child center on?

21. How do you celebrate religious holidays in your home?

22. How many of the conflicts in your marriage are due directly or indirectly to religious differences between you and what have you done to manage those conflicts specifically?

23. Were there any unforeseen obstacles you had to overcome, if so, how did you overcome these challenges?

24. What are some challenges or harms that you feel religion could cause?

25. As individuals and as a couple you have built a successful marriage. If you could share any marriage related counsel or advice, or insight with unmarried young adults, what would it be? What would you tell someone else who is entering into an interfaith marriage?

**Quantitative Comparisons Between Continuously-Interfaith and Divergent-Interfaith Couples**

We explored differences in the challenges and adaptive processes between continuously-interfaith and divergent-interfaith couples using t-tests and Fisher’s exact test due to the lack of research comparing these two groups (see Table S2 – S4). Using NVivo’s matrix coding function, we estimated the frequency for each couple reporting each theme and exported the results. Then, in Stata 17, we created a binary variable to distinguish continuously-interfaith couples from divergent-interfaith and compared the means of the two groups on the frequency they reported each theme using independent sample t-tests. Since a couple discussing a theme more frequently does not necessarily imply they engage in it more often in their marriage, we also created categorical variables indicating whether each couple discussed each theme at all. Given the small sample size, we used Fisher’s exact test instead of the chi-square test to examine the significance of the association between group membership and theme reporting (Kim, 2017). Results partially indicated that divergent-interfaith couples discussed more marital challenges, the importance of fostering religious intimacy, and respect for each other’s religious choices, and emphasized making marriage their highest priority more than continuously-interfaith couples.

**Table S2**

| *Frequencies of References Made by Continuously-interfaith and Divergent-interfaith Couples* | | | | | |
| --- | --- | --- | --- | --- | --- |
| Theme |  | # of Continuously-Interfaith References (%) | | # of Divergent-Interfaith References (%) | |
| Marital Challenges in Navigating Religious Differences | | | 51/107 (48%) | | 56/107 (52%) |
| A. **A**ssist and Support Each Other’s Practices and Beliefs | | | 117/143 (82%) | | 26/143 (18%) |
| F. **F**ocus on Similarities of Belief | | | 102/129 (79%) | | 27/129 (21%) |
| F.I. **F**oster Religious **I**ntimacy and Understanding | | | 60/94 (64%) | | 34/94 (36%) |
| R. **R**espect Each Other’s Religious Choices | | | 86/126 (68%) | | 40/126 (32%) |
| M. Make **M**arriage the First and Highest Priority | | | 7/21 (33%) | | 14/21 (67%) |
|  | # Total | | 25 | | 5 |

# Table S3

| *T-tests of Theme Frequency Between Continuously-interfaith and Divergent-interfaith Couples* | | | | | |  |  |  |
| --- | --- | --- | --- | --- | --- | --- | --- | --- |
| Theme |  | Continuously-interfaith  *M (SD)* | | Divergent-Interfaith  *M (SD)* | | *T* | *p* | *Cohen’s d* |
| Marital Challenges in Navigating Religious Differences | | | **2.04 (2.70)*** | **11.20 (6.42)** | | **–5.37** | **< .001** | **-2.63** |
| A. **A**ssist and Support Each Other’s Practices and Beliefs | | | 4.68 (4.26) | | 5.20 (2.59) | –0.26 | .40 | -.13 |
| F. **F**ocus on Similarities of Belief | | | 4.08 (2.91) | | 5.40 (2.70) | –0.93 | .36 | -.46 |
| F.I. **F**oster Religious **I**ntimacy and Understanding | | | **2.40 (2.40)*** | | **6.80 (3.90)** | **–3.37** | **.002** | **-1.65** |
| R. **R**espect Each Other’s Religious Choices | | | **3.44 (3.18)*** | | **8.00 (4.58)** | **–2.72** | **.011** | **-1.34** |
| M. Make **M**arriage the First and Highest Priority | | | **0.28 (0.61)*** | | **2.80 (2.77)** | **–4.31** | **<.001** | **-2.11** |

*Note.* Means and analyses are estimated at the couple level.

**p <* .05. *df* (28).

# Table S4

*Frequencies of Interfaith and Divergent-interfaith Couples Reporting Each Theme and Results of Fisher’s Exact Test*

| Theme |  | # of Continuously-interfaith Couples (%) | | | # of Divergent-Interfaith Couples (%) | *p* | *Cramer's V* |
| --- | --- | --- | --- | --- | --- | --- | --- |
| Marital Challenges in Navigating Religious Differences | | | 15/25 (63%) | 5/5 (100%) | | .14 | .32 |
| A. **A**ssist and Support Each Other’s Practices and Beliefs | | | 23/25 (92%) | | 5/5 (100%) | 1.00 | .12 |
| F. **F**ocus on Similarities of Belief | | | 24/25 (96%) | | 5/5 (100%) | 1.00 | .08 |
| F.I. **F**oster Religious **I**ntimacy and Understanding | | | 19/25 (76%) | | 5/5 (100%) | .55 | .22 |
| R. **R**espect Each Other’s Religious Choices | | | 21/25 (84%) | | 5/5 (100%) | 1.00 | .16 |
| M. Make **M**arriage the First and Highest Priority | | | **5/25 (20%)*** | | **4/5 (80%)** | **.019** | **.40** |

*Note.* **p <* .05.

**References**

Kim, H. Y. (2017). Statistical notes for clinical researchers: Chi-squared test and Fisher's exact test. *Restorative Dentistry & Endodontics*, *42*(2), 152-155.
